# Crystal structure of 2,2-di­phenyl­hydrazinium chloride

**DOI:** 10.1107/S1600536814022879

**Published:** 2014-10-24

**Authors:** Amit Kumal Paul, Soma Mukherjee, Helen Stoeckli-Evans

**Affiliations:** a Department of Environmental Science, University of Kalyani, Kalyani, Nadia 741 235, West Bengal, India; b Institute of Physics, University of Neuchâtel, rue Emile-Argand 11, CH-2000 Neuchâtel, Switzerland

**Keywords:** crystal structure, di­phenyl­hydrazine, hydrazinium, hydrogen bonding

## Abstract

In the title compound, C_12_H_13_N_2_^+^·Cl^−^, the chloride salt of 1,1′-di­phenyl­hydrazine, the phenyl rings are inclined to one another by 78.63 (17)°. The N—^+^NH_3_ bond lengths is 1.445 (3) Å, and the N—C_phen­yl_ bond lengths are 1.435 (3) and 1.447 (4) Å. In the crystal, mol­ecules are linked *via* N—H⋯Cl hydrogen bonds, forming chains along [10-1], which enclose two adjacent *R* ^4^_2_(6) ring motifs. The chains are reinforced by C—H⋯Cl hydrogen bonds.

## Abstract 2

In the title mol­ecule, the phenyl rings are inclined to one another by 78.63 (17)°. In the crystal, mol­ecules are linked *via* N—H⋯Cl hydrogen bonds, forming chains along [10-1], which enclose two adjacent 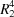
(6) ring motifs.

## Chemical context   

1,1′-Di­phenyl­hydrazine is a ‘free’ hydrazine, *viz* with an NH_2_ group. It has been used as a starting reagent for the preparation of Schiff bases as fluorescent sensors for fluoride (Mukherjee *et al.*, 2014 ▶), and metal complexes (Stender *et al.*, 2003 ▶; Clulow *et al.*, 2008 ▶). The title compound, (I), crystallized out of a reaction of 1,1′-di­phenyl­hydrazine with 2,6-di­acetyl­pyridine in an attempt to prepare the ligand 2,6-di­acetyl­pyridine bis­(*N*,*N*-di­phenyl­hydrazone). The latter compound is one of a series that has been used to prepare bis­(imino)­pyridyl iron and cobalt complexes to study the effect of nitro­gen substituents on ethyl­ene oligomerization and polymerization (Britovsek *et al.*, 2001 ▶).
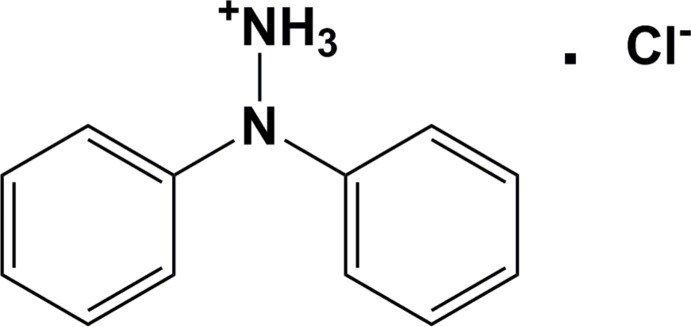


## Structural commentary   

**Figure 1:**
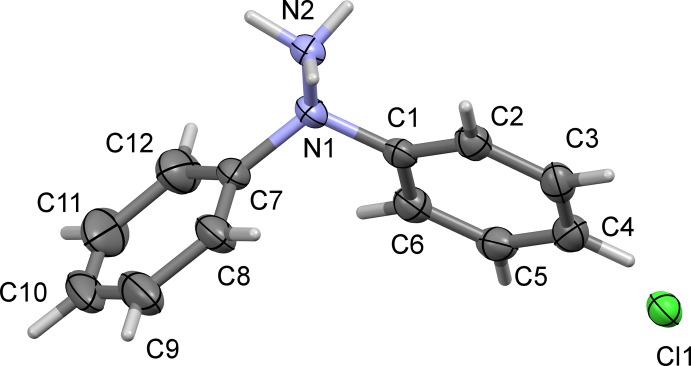
A view of the mol­ecular structure of the title compound with atom labelling. Displacement ellipsoids are drawn at the 50% probability level.

The mol­ecular structure of the title salt, (I), is illustrated in Fig. 1 ▶, and selected bond distances and bond angles are given in Table 1 ▶. The two phenyl rings (C1–C6 and C7–C12) are inclined to one another by 78.63 (17)°. The N1—N2 bond lengths is 1.445 (3) Å and the N1—C1 and N1—C7 bond lengths are 1.435 (3) and 1.447 (4) Å, respectively. Atom N1 is displaced from the plane of the three connected atoms, (N2/C1/C7), by 0.370 (2) Å, while the sum of the three angles involving atom N1 is 340.9 °. This illustrates clearly the pyramidal nature of the central N atom, N1.

## Supra­molecular features   

**Figure 2:**
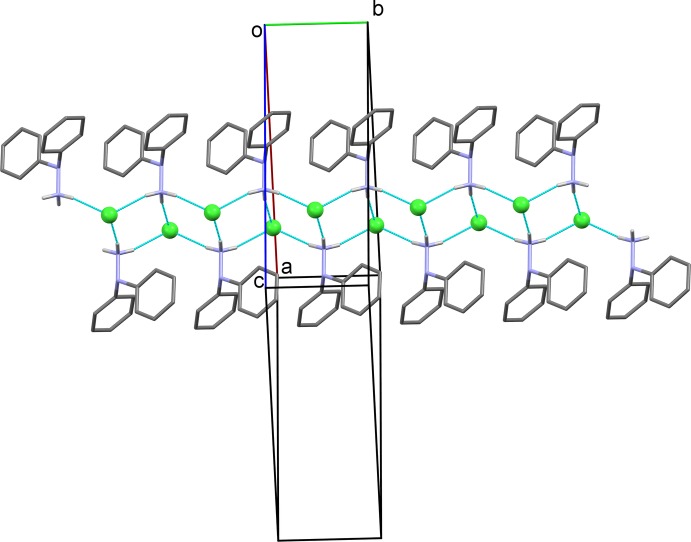
A partial view normal to (10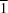) of the crystal packing of the title compound. Hydrogen bonds are shown as dashed lines (see Table 2 ▶ for details; C-bound H atoms have been omitted for clarity).

**Figure 3:**
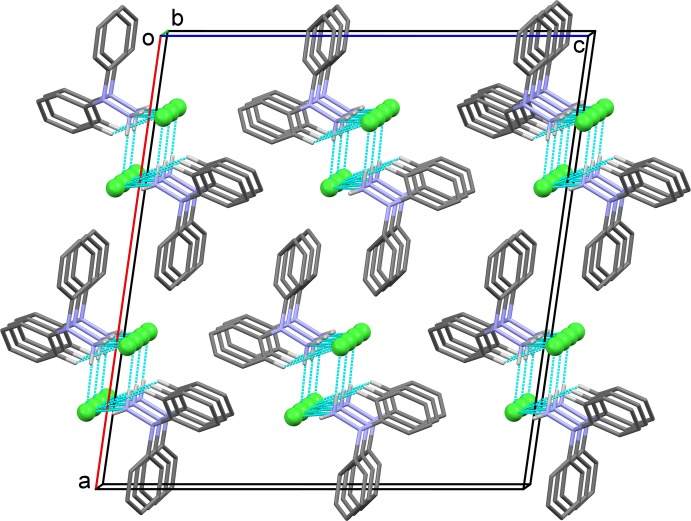
A view along the *b* axis of the crystal packing of the title compound. Hydrogen bonds are shown as dashed lines (see Table 2 ▶ for details; C-bound H atoms not involved in hydrogen bonding have been omitted for clarity).

In the crystal of compound (I), mol­ecules are linked *via* N—H⋯Cl hydrogen bonds, forming chains along [10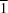], which enclose two adjacent 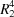
(6) ring motifs (Table 2 ▶ and Fig. 2 ▶). The chains are reinforced by C—H⋯Cl hydrogen bonds (Fig. 3 ▶ and Table 2 ▶).

## Database survey   

A search of the Cambridge Structural Database (Version 5.35, last update May 2014; Groom & Allen, 2014 ▶) yielded only two hits for the sub-structure 1,1′-di­phenyl­hydrazine: *viz*. 1,1′-di­phenyl­hydrazinium di­cyano­gold(I) monohydrate (II) (Stender *et al.*, 2003 ▶) and 1,1′-di­phenyl­hydrazine (III) itself (Clulow *et al.*, 2008 ▶).

The structure of salt (II) is very similar to that of the title compound, (I). The two phenyl rings are inclined to one another by 80.04 (19)° compared to 78.63 (17)° in (I). The bond lengths and angles involving the central N atom are also very similar to those in (I). The central N atom is displaced by 0.358 (3) Å from the plane of the three attached N and C atoms, and the sum of their bond angles is 342.0°, indicating clearly the pyramidal nature of the central N atom, as in (I).

In 1,1′-di­phenyl­hydrazine (III), which crystallized with two independent mol­ecules per asymmetric unit, the phenyl rings are inclined to one another by only 58.39 (2) and 52.30 (9)°, and the N—NH_2_ bond lengths are 1.418 (2) and 1.411 (3) Å. The central N atoms are displaced by 0.1199 (17) and 0.0828 (19) Å from the planes of the three attached N and C atoms, with the sums of their bond angles being 357.85 and 358.97°. This confirms the trigonal–planar conformation of the central N atom.

In the crystal of compound (II), mol­ecules are linked by N—H⋯N, N—H⋯O and O—H⋯N hydrogen bonds, forming two-dimensional networks parallel to (001). These sheets are linked *via* C—H⋯π inter­actions, forming a three-dimensional structure. In the crystal of compound (III), there are no hydrogen bonds present with only weak C—H⋯π inter­actions linking the mol­ecules to form chains along [100]. There are no π–π inter­actions present in the crystal structures of any of the three compounds.

## Synthesis and crystallization   

Brown block-like crystals of the title compound were obtained during an attempt to prepare the ligand 2,6-di­acetyl­pyridine bis­(*N*,*N*-di­phenyl­hydrazone) by a condensation reaction involving 1,1′-di­phenyl­hydrazinium hydro­chloride and 2,6-di­acetyl­pyridine in methanol.

## Refinement details   

Crystal data, data collection and structure refinement details are summarized in Table 3 ▶. The ammonium H atoms were located in a difference Fourier map and freely refined. The C-bound H atoms were included in calculated positions and treated as riding atoms: C—H = 0.95 Å with *U*_iso_(H) = 1.2*U*_eq_(C).

## Supplementary Material

Crystal structure: contains datablock(s) I, global. DOI: 10.1107/S1600536814022879/lh5735sup1.cif

Structure factors: contains datablock(s) I. DOI: 10.1107/S1600536814022879/lh5735Isup2.hkl

Supporting information file. DOI: 10.1107/S1600536814022879/lh5735Isup3.cml

CCDC reference: 1029761

Additional supporting information:  crystallographic information; 3D view; checkCIF report

## Figures and Tables

**Table 1:** Selected geometric parameters (, )

N1N2	1.445(3)	N1C7	1.447(4)
N1C1	1.435(3)		
			
C1N1N2	113.4(2)	N2N1C7	111.5(2)
C1N1C7	116.0(2)		

**Table 2:** Hydrogen-bond geometry (, )

*D*H*A*	*D*H	H*A*	*D* *A*	*D*H*A*
N2H1*N*Cl1^i^	0.92(3)	2.31(3)	3.208(3)	165(3)
N2H2*N*Cl1^ii^	0.96(3)	2.23(3)	3.167(3)	167(3)
N2H3*N*Cl1^iii^	0.86(4)	2.30(4)	3.154(3)	175(3)
C2H2Cl1^i^	0.95	2.96	3.696(3)	135

Symmetry codes: (i) 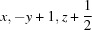; (ii) 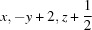; (iii) 
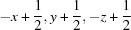
.

**Table 3:** Experimental details

Crystal data	
Chemical formula	C_12_H_13_N_2_^+^Cl
*M*_r_	220.69
Crystal system, space group	Monoclinic, *C*2/*c*
Temperature (K)	173
*a*, *b*, *c* ()	21.341(3), 5.3728(4), 19.940(3)
()	98.291(10)
*V* (^3^)	2262.4(5)
*Z*	8
Radiation type	Mo *K*
(mm^1^)	0.31
Crystal size (mm)	0.45 0.35 0.25

Data collection	
Diffractometer	STOE IPDS 2
Absorption correction	Multi-scan (*MULscanABS* in *PLATON*; Spek, 2009 ▶)
*T*_min_, *T*_max_	0.578, 1.000
No. of measured, independent and observed [*I* > 2(*I*)] reflections	7392, 2140, 1517
*R*_int_	0.120
(sin /)_max_ (^1^)	0.609

Refinement	
*R*[*F* ^2^ > 2(*F* ^2^)], *wR*(*F* ^2^), *S*	0.057, 0.141, 0.93
No. of reflections	2140
No. of parameters	148
H-atom treatment	H atoms treated by a mixture of independent and constrained refinement
_max_, _min_ (e ^3^)	0.30, 0.47

Computer programs: *X-AREA* and *X-RED32* (Stoe Cie, 2009 ▶), *SHELXS2013* and *SHELXL2013* (Sheldrick, 2008 ▶), *PLATON* (Spek, 2009 ▶), *Mercury* (Macrae *et al.*, 2008 ▶), and *publCIF* (Westrip, 2010 ▶).

## supplementary crystallographic information

### Crystal data

**Table:**

C_12_H_13_N_2_^+^·Cl^−^	*F*(000) = 928
*M**_r_* = 220.69	*D*_x_ = 1.296 Mg m^−^^3^
Monoclinic, *C*2/*c*	Mo *K*α radiation, λ = 0.71073 Å
*a* = 21.341 (3) Å	Cell parameters from 5046 reflections
*b* = 5.3728 (4) Å	θ = 1.9–26.0°
*c* = 19.940 (3) Å	µ = 0.31 mm^−^^1^
β = 98.291 (10)°	*T* = 173 K
*V* = 2262.4 (5) Å^3^	Block, brown
*Z* = 8	0.45 × 0.35 × 0.25 mm

### Data collection

**Table:**

STOE IPDS 2 diffractometer	2140 independent reflections
Radiation source: fine-focus sealed tube	1517 reflections with *I* > 2σ(*I*)
Plane graphite monochromator	*R*_int_ = 0.120
φ + ω scans	θ_max_ = 25.6°, θ_min_ = 1.9°
Absorption correction: multi-scan (*MULscanABS* in *PLATON*; Spek, 2009)	*h* = −24→25
*T*_min_ = 0.578, *T*_max_ = 1.000	*k* = −6→5
7392 measured reflections	*l* = −24→24

### Refinement

**Table:**

Refinement on *F*^2^	Primary atom site location: structure-invariant direct methods
Least-squares matrix: full	Secondary atom site location: difference Fourier map
*R*[*F*^2^ > 2σ(*F*^2^)] = 0.057	Hydrogen site location: mixed
*wR*(*F*^2^) = 0.141	H atoms treated by a mixture of independent and constrained refinement
*S* = 0.93	*w* = 1/[σ^2^(*F*_o_^2^) + (0.0794*P*)^2^] where *P* = (*F*_o_^2^ + 2*F*_c_^2^)/3
2140 reflections	(Δ/σ)_max_ < 0.001
148 parameters	Δρ_max_ = 0.30 e Å^−^^3^
0 restraints	Δρ_min_ = −0.47 e Å^−^^3^

### Special details

**Table:**

Geometry. All e.s.d.'s (except the e.s.d. in the dihedral angle between two l.s. planes) are estimated using the full covariance matrix. The cell e.s.d.'s are taken into account individually in the estimation of e.s.d.'s in distances, angles and torsion angles; correlations between e.s.d.'s in cell parameters are only used when they are defined by crystal symmetry. An approximate (isotropic) treatment of cell e.s.d.'s is used for estimating e.s.d.'s involving l.s. planes.

### Fractional atomic coordinates and isotropic or equivalent isotropic displacement parameters (Å^2^)

**Table:**

	*x*	*y*	*z*	*U*_iso_*/*U*_eq_	
N1	0.14173 (10)	0.9765 (4)	0.38714 (11)	0.0284 (5)	
N2	0.17735 (12)	0.9687 (5)	0.45449 (12)	0.0296 (5)	
H1N	0.1692 (14)	0.834 (6)	0.4807 (14)	0.032 (8)*	
H2N	0.1695 (15)	1.113 (6)	0.4803 (14)	0.034 (8)*	
H3N	0.217 (2)	0.982 (7)	0.4537 (17)	0.046 (10)*	
C1	0.15920 (13)	0.7846 (5)	0.34332 (13)	0.0282 (6)	
C2	0.20210 (14)	0.5967 (5)	0.36467 (14)	0.0312 (6)	
H2	0.2207	0.5880	0.4108	0.037*	
C3	0.21793 (14)	0.4209 (5)	0.31865 (14)	0.0345 (7)	
H3	0.2470	0.2917	0.3336	0.041*	
C4	0.19146 (15)	0.4339 (6)	0.25115 (15)	0.0376 (7)	
H4	0.2025	0.3151	0.2196	0.045*	
C5	0.14855 (14)	0.6226 (6)	0.23008 (14)	0.0357 (7)	
H5	0.1301	0.6317	0.1838	0.043*	
C6	0.13236 (14)	0.7966 (5)	0.27525 (14)	0.0336 (6)	
H6	0.1030	0.9246	0.2601	0.040*	
C7	0.07462 (14)	1.0052 (5)	0.38918 (15)	0.0334 (7)	
C8	0.04095 (15)	0.8348 (7)	0.42154 (16)	0.0438 (8)	
H8	0.0617	0.6955	0.4442	0.053*	
C9	−0.02370 (18)	0.8694 (9)	0.42060 (19)	0.0622 (11)	
H9	−0.0474	0.7536	0.4427	0.075*	
C10	−0.05343 (18)	1.0729 (10)	0.3874 (2)	0.0708 (14)	
H10	−0.0976	1.0975	0.3870	0.085*	
C11	−0.0194 (2)	1.2384 (9)	0.3551 (3)	0.0798 (15)	
H11	−0.0405	1.3756	0.3317	0.096*	
C12	0.04547 (17)	1.2096 (7)	0.3560 (2)	0.0561 (10)	
H12	0.0691	1.3272	0.3344	0.067*	
Cl1	0.17516 (3)	0.53583 (13)	0.04013 (3)	0.0316 (2)	

### Atomic displacement parameters (Å^2^)

**Table:**

	*U*^11^	*U*^22^	*U*^33^	*U*^12^	*U*^13^	*U*^23^
N1	0.0175 (11)	0.0303 (12)	0.0360 (12)	0.0013 (9)	−0.0004 (9)	−0.0032 (9)
N2	0.0203 (13)	0.0317 (13)	0.0361 (13)	−0.0011 (11)	0.0012 (10)	−0.0047 (11)
C1	0.0192 (14)	0.0282 (14)	0.0376 (14)	−0.0040 (11)	0.0052 (11)	−0.0018 (11)
C2	0.0271 (15)	0.0305 (15)	0.0363 (14)	−0.0017 (12)	0.0055 (12)	0.0028 (11)
C3	0.0308 (17)	0.0290 (15)	0.0460 (16)	0.0025 (12)	0.0128 (13)	0.0030 (12)
C4	0.0348 (18)	0.0366 (16)	0.0441 (16)	−0.0056 (14)	0.0148 (13)	−0.0082 (13)
C5	0.0291 (16)	0.0414 (16)	0.0363 (15)	−0.0084 (13)	0.0039 (12)	−0.0036 (12)
C6	0.0255 (15)	0.0348 (15)	0.0401 (15)	−0.0010 (12)	0.0036 (12)	0.0034 (12)
C7	0.0203 (14)	0.0348 (16)	0.0433 (15)	0.0017 (12)	−0.0013 (11)	−0.0123 (12)
C8	0.0226 (16)	0.057 (2)	0.0510 (18)	−0.0017 (15)	0.0043 (13)	−0.0060 (15)
C9	0.031 (2)	0.096 (3)	0.062 (2)	−0.008 (2)	0.0132 (17)	−0.025 (2)
C10	0.0222 (19)	0.102 (4)	0.084 (3)	0.008 (2)	−0.0050 (18)	−0.050 (3)
C11	0.041 (2)	0.067 (3)	0.120 (4)	0.024 (2)	−0.026 (2)	−0.027 (3)
C12	0.036 (2)	0.045 (2)	0.082 (3)	0.0090 (16)	−0.0091 (17)	−0.0038 (18)
Cl1	0.0232 (4)	0.0334 (4)	0.0381 (4)	0.0010 (3)	0.0044 (3)	0.0008 (3)

### Geometric parameters (Å, º)

**Table:**

N1—N2	1.445 (3)	C5—C6	1.376 (4)
N1—C1	1.435 (3)	C5—H5	0.9500
N1—C7	1.447 (4)	C6—H6	0.9500
N2—H1N	0.92 (3)	C7—C8	1.380 (5)
N2—H2N	0.96 (3)	C7—C12	1.383 (4)
N2—H3N	0.86 (4)	C8—C9	1.390 (5)
C1—C2	1.388 (4)	C8—H8	0.9500
C1—C6	1.397 (4)	C9—C10	1.384 (7)
C2—C3	1.392 (4)	C9—H9	0.9500
C2—H2	0.9500	C10—C11	1.365 (7)
C3—C4	1.384 (4)	C10—H10	0.9500
C3—H3	0.9500	C11—C12	1.392 (6)
C4—C5	1.390 (4)	C11—H11	0.9500
C4—H4	0.9500	C12—H12	0.9500
			
C1—N1—N2	113.4 (2)	C4—C5—H5	119.5
C1—N1—C7	116.0 (2)	C5—C6—C1	119.9 (3)
N2—N1—C7	111.5 (2)	C5—C6—H6	120.1
N1—N2—H1N	115.5 (19)	C1—C6—H6	120.1
N1—N2—H2N	111.5 (18)	C8—C7—C12	121.5 (3)
H1N—N2—H2N	106 (3)	C8—C7—N1	121.8 (3)
N1—N2—H3N	112 (2)	C12—C7—N1	116.8 (3)
H1N—N2—H3N	110 (3)	C7—C8—C9	119.2 (4)
H2N—N2—H3N	101 (3)	C7—C8—H8	120.4
C2—C1—C6	119.5 (3)	C9—C8—H8	120.4
C2—C1—N1	123.6 (2)	C10—C9—C8	119.9 (4)
C6—C1—N1	116.9 (2)	C10—C9—H9	120.1
C1—C2—C3	120.2 (3)	C8—C9—H9	120.1
C1—C2—H2	119.9	C11—C10—C9	120.1 (4)
C3—C2—H2	119.9	C11—C10—H10	119.9
C4—C3—C2	120.2 (3)	C9—C10—H10	119.9
C4—C3—H3	119.9	C10—C11—C12	121.2 (4)
C2—C3—H3	119.9	C10—C11—H11	119.4
C3—C4—C5	119.3 (3)	C12—C11—H11	119.4
C3—C4—H4	120.4	C7—C12—C11	118.2 (4)
C5—C4—H4	120.4	C7—C12—H12	120.9
C6—C5—C4	120.9 (3)	C11—C12—H12	120.9
C6—C5—H5	119.5		
			
N2—N1—C1—C2	5.2 (4)	C1—N1—C7—C8	72.9 (3)
C7—N1—C1—C2	−125.8 (3)	N2—N1—C7—C8	−59.0 (3)
N2—N1—C1—C6	−172.7 (2)	C1—N1—C7—C12	−105.9 (3)
C7—N1—C1—C6	56.3 (3)	N2—N1—C7—C12	122.2 (3)
C6—C1—C2—C3	−0.4 (4)	C12—C7—C8—C9	0.1 (5)
N1—C1—C2—C3	−178.2 (2)	N1—C7—C8—C9	−178.7 (3)
C1—C2—C3—C4	0.7 (4)	C7—C8—C9—C10	0.1 (5)
C2—C3—C4—C5	−0.6 (4)	C8—C9—C10—C11	0.5 (6)
C3—C4—C5—C6	0.3 (4)	C9—C10—C11—C12	−1.3 (6)
C4—C5—C6—C1	0.0 (4)	C8—C7—C12—C11	−0.8 (5)
C2—C1—C6—C5	0.1 (4)	N1—C7—C12—C11	178.0 (3)
N1—C1—C6—C5	178.1 (2)	C10—C11—C12—C7	1.4 (6)

### Hydrogen-bond geometry (Å, º)

**Table:**

*D*—H···*A*	*D*—H	H···*A*	*D*···*A*	*D*—H···*A*
N2—H1*N*···Cl1^i^	0.92 (3)	2.31 (3)	3.208 (3)	165 (3)
N2—H2*N*···Cl1^ii^	0.96 (3)	2.23 (3)	3.167 (3)	167 (3)
N2—H3*N*···Cl1^iii^	0.86 (4)	2.30 (4)	3.154 (3)	175 (3)
C2—H2···Cl1^i^	0.95	2.96	3.696 (3)	135

Symmetry codes: (i) *x*, −*y*+1, *z*+1/2; (ii) *x*, −*y*+2, *z*+1/2; (iii) −*x*+1/2, *y*+1/2, −*z*+1/2.

## References

[bb1] Britovsek, G. J. P., Gibson, V. C., Kimberley, B. S., Mastroianni, S., Redshaw, C., Solan, G. A., White, A. J. P. & Williams, D. J. (2001). *J. Chem. Soc. Dalton Trans.* pp. 1639–1644.

[bb2] Clulow, A. J., Selby, J. D., Cushion, M. G., Schwarz, A. D. & Mountford, P. (2008). *Inorg. Chem.* **47**, 12049–12062. 10.1021/ic801735c 18998672

[bb3] Groom, C. R. & Allen, F. H. (2014). *Angew. Chem. Int. Ed.* **53**, 662–671. 10.1002/anie.201306438 24382699

[bb4] Macrae, C. F., Bruno, I. J., Chisholm, J. A., Edgington, P. R., McCabe, P., Pidcock, E., Rodriguez-Monge, L., Taylor, R., van de Streek, J. & Wood, P. A. (2008). *J. Appl. Cryst.* **41**, 466–470.

[bb5] Mukherjee, S., Paul, A. K. & Stoeckli-Evans, H. (2014). *Sens. Actuators B Chem.* **202**, 1190–1199.

[bb6] Sheldrick, G. M. (2008). *Acta Cryst.* A**64**, 112–122. 10.1107/S0108767307043930 18156677

[bb7] Spek, A. L. (2009). *Acta Cryst.* D**65**, 148–155. 10.1107/S090744490804362X PMC2631630 19171970

[bb8] Stender, M., Olmstead, M. M., Balch, A. L., Rios, D. & Attar, S. (2003). *Dalton Trans.* pp. 4282–4287.

[bb9] Stoe & Cie. (2009). *X-AREA* and *X-RED32*. Stoe & Cie GmbH, Darmstadt, Germany.

[bb10] Westrip, S. P. (2010). *J. Appl. Cryst.* **43**, 920–925.

